# Antibiotic-loaded bone cement combined with vacuum sealing drainage to treat deep sternal wound infection following cardiac surgery: the first case report

**DOI:** 10.1186/s13019-021-01673-x

**Published:** 2021-10-10

**Authors:** Xia Jiang, Fanyu Bu, Yong Xu, Zhaohui Jing, Guoqing Jiao, Mingqiu Li, Xiaosong Rong

**Affiliations:** 1 Department of Cardiovascular Surgery, Wuxi People’s Hospital/Wuxi Affiliated Hospital of Nanjing Medical University, Wuxi, 214203 China; 2 Department of Chronic Wound, Wuxi Ninth People’s Hospital Affiliated to Soochow University, Wuxi, 214062 China

**Keywords:** Antibiotic-loaded bone cement, Vacuum sealing drainage, Deep sternal wound infection

## Abstract

**Background:**

Deep sternal wound infection (DSWI) is a rare but serious complication after median sternotomy, and treatment success depends mainly on surgical experience. Here we first present a case of a patient successfully treated for antibiotic-loaded bone cement (ALBC) combined with vacuum sealing drainage (VSD) of DSWI.

**Case presentation:**

This case report presented a patient who underwent open heart surgery, and suffered postoperatively from a DSWI associated with enterococcus cloacae. Focus debridement combined with ALBC filling and VSD was conducted in stage I. Appropriate antibiotics were started according to sensitivity to be continued for 2 weeks until the inflammatory markers decreased to normal. One month after the surgery, patient’s wound was almost healed and was discharged from hospital with a drainage tube. Two months after the stage I surgery procedure, the major step was removing the previous ALBC, and extensive debridement in stage II. The patient fully recovered without further surgical treatment.

**Conclusions:**

The results of this case suggest that ALBC combined with VSD may be a viable and safe option for deep sternal wound reconstruction.

## Background

Deep sternal wound infection (DSWI) is a rare but potentially devastating complication of median sternotomy performed in cardiac surgery. The incidence of this complication ranges between 1 and 3% [1, 2] and on average mortality of 10–47% [3, 4]. It is difficult to treat when compared to skin and subcutaneous tissue infections. We herein report a successful treatment of DSWI after open heart surgery with ALBC and VSD.

## Case presentation

**Fig. 1:**
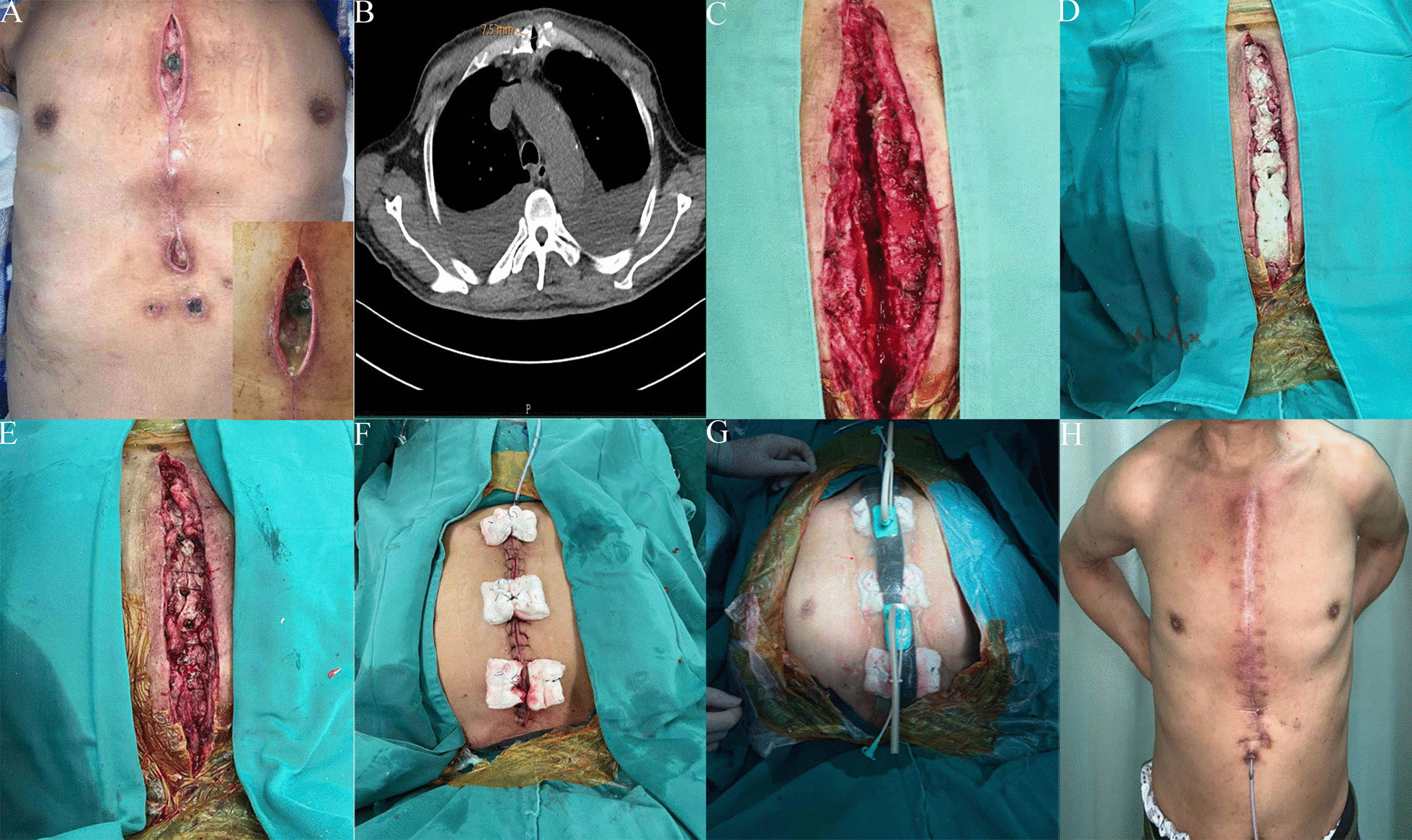
I-stage surgical procedure of the DSWI. **a** Post-sternotomy wound infection; **b** Chest CT revealed sternal dehiscence and non-unionc; **c** The wound after debridement; **d** ALBC was inseted on the sternal defect; **e** Mobilization of pectoralis major muscle flap and intermittent sutures; **f** Deep sternal wound infection-associated defect after reconstruction and skin suture; **g** VSD coverage; **h** 4th postoperative week

Here we report the case of a 53-year old male patient, who underwent mitral valve replacement and coronary artery bypass surgery (LIMA to LADA) on April 16, 2019. He was a known case of diabetes and chronic obstructive pulmonary disease (COPD). Five weeks after surgery, the patient was referred to our center with a 1-week history of abundant discharge accompanied by fever (39.5 °C), painful sternal instability, and shortness of breath. He had a purulent wound in the upper part of his sternotomy incision, with a fistula approximately 4 cm long (Fig. 1a). A computed tomography (CT) scan of the thorax conducted in response showed sternal non-union up to 7 mm wide (Fig. 1b). Inflammatory markers were significant with a white blood cell count of 19.5 × 10^9^/L and an erythrocyte sedimentation rate (ESR) of 77 mm/H. A C-reactive protein (CRP) level was 105.9 mg/L. Serum albumin and hemoglobin were 28 g/L and 85 g/L, respectively. The wound culture examination revealed vancomycin-sensitive enterococcus cloacae. DSWI with sternal dehiscence was the diagnosis. An operation was scheduled immediately because of severe infection symptoms on May 28, 2019. General anesthesia was administered to this patient prior to surgery. The surgical technique was divided in two stages. Stage I: (1) aggressive debridement was performed on this patient. All abnormally proliferated granulation tissue and residual foreign bodies were removed. Then, the wound was rinsed repeatedly with hydrogen peroxide, iodophor, and normal saline (Fig. 1c). (2) Antibiotic impregnated cement (PALACOS MVⓇ + G bone cement, Heraeus, Heraeus Medical GmbH, Wehrheim, Germany) was prepared by combining a 40 g bag of cement with 2 g of vancomycin. The sternal defect was filled with an appropriate amount of ALBC, which provided a reliable bone coverage (Fig. 1d). Also, the holes were made on the surface of ALBC for drainage. (3) Next, bilateral pectoralis major muscle flaps and subcutaneous tissue were raised off, from the chest wall to adistance of about 4 cm from incision margin (Fig. 1e). Furthermore, the drainage tube was placed between ALBC and the subcutaneous layer. And then, the skin was relaxedly sutured without significant tension (Fig. 1f). Finally, the skin around the wound was cleaned with 75% alcohol and a semipermeable membrane was used to seal the wound and the VSD (Wu han VSD Medical Science & Technology Co., Ltd. Vacuum Sealing Drainage Dressing VSD-D-2-15*10 cm) dressing (Fig. 1g), the negative pressure is −75 mmHg to −100 mmHg. The VSD was not changed during the treatment, and it was removed after intermittent use for 1 week. The drainage-fluid culture (tested twice), ESR, and CRP were normal after 10 days of intravenous vancomycin antibiotics therapy. The thoracic cage was stable and he was symptom-free (Fig. 1h).

**Fig. 2:**
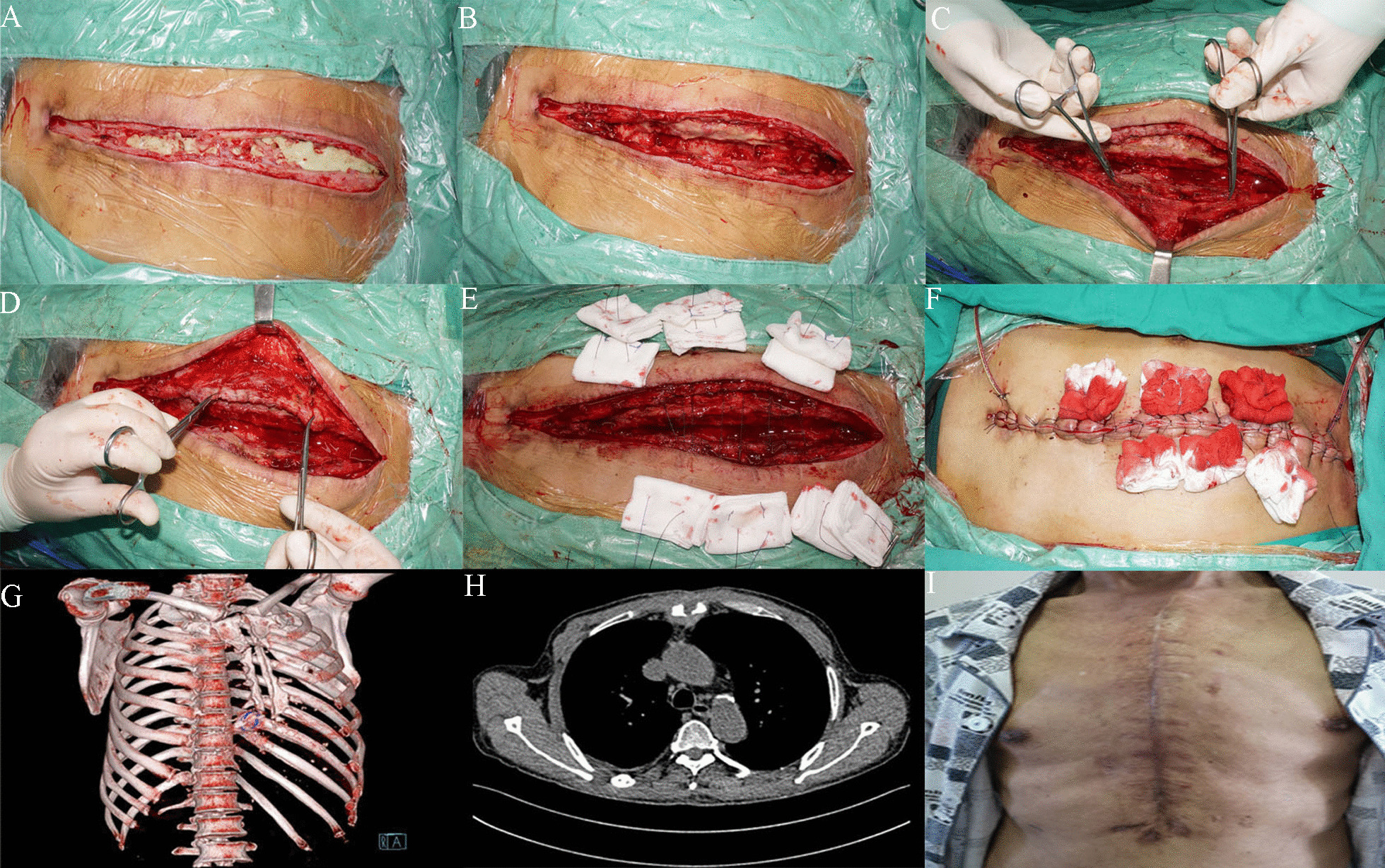
II-stage surgical procedure of the DSWI. **a** ALBC covering the surface of sternal; **b** The wound after removing the retained ALBC and complete debridement; **c** Mobilization of left pectoralis major muscle flap; **d** Mobilization of right pectoralis major muscle flap; **e** The subcutaneous tissue and pectoralis major muscle flap were intermittently sutured by methods of relieving tension and no residual foreign bodies; **f** Drain tubes were placed: one under the muscle flap and the other under the subcutaneous layer; **g** Three-dimensional rib reconstruction revealed sternal dehiscence; **h** CT image of 3 months after final surgical reconstruction; **i** Healed sternal wound at the 3-month postoperative visit

Two months after the stage I surgery procedure, our final step was removing the previous ALBC (Fig. 2a), and extensive debridement in stage II. The second re-exploration revealed a clean, red, granulating wound bed was achieved (Fig. 2b). Subsequently, the bilateral pectoralis major muscle flap were mobilized from the thorax wall again (Fig. 2c, d). The subcutaneous tissue and bilateral pectoralis major muscle flap were intermittently sutured to cover sternum defect by methods of relieving tension and no residual foreign bodies (Fig. 2e). Furthermore, two drain tubes were placed: one under the muscle flap and the other under the subcutaneous layer (Fig. 2f). Sutures were removed 14 days after the operation and this patient was discharged in good local and general condition on August 17, 2019, 20 days after the stage II surgery. Drainage tubes were removed when output was less than 5 ml/day for 3 days. Three months postoperatively, the skin healed nicely (Fig. 2I), and three-dimensional rib reconstruction revealed sternal dehiscence as before (Fig. 2g). A final CT scan documented scar tissue covering the mediastinum (Fig. 2h). More than 1 years after this surgery, the patient did not relapse.

## Discussion and conclusions

One of the life-threatening complications that occurs in patients following cardiovascular surgery through median sternotomy is DSWI. As defined by the Centers for Disease Control and Prevention [5], DSWI diagnosis requires at least one of the following criteria: (I) an organism is isolated from culture of mediastinal tissue or fluid; (II) evidence of mediastinitis seen during surgery; (III) one of the following conditions: chest pain, sternal instability, or fever (> 38 °C) in combination with either purulent discharge from the mediastinum or isolation of an organism from culture of blood or mediastinal drainage. This case was diagnosed with DSWI by an attending physician and an chronic wound control physician during his hospital stay period based on the definition of DSWI. Many authors have investigated the aetiology of DSWI in the past decades. Patient-related risk factors include age, diabetes, renal failure, smoking, obesity, and COPD [6, 7]. Risk factors during surgery are internal mammary artery use (decreased sternal bone blood flow), increasing number of grafts, re-exploration for bleeding, blood product usage [8–10]. Current treatment comprises antibiotics, debridement, VSD wound therapy and sometimes transposition of muscle or omental flaps to fill the anterior mediastinal dead space. Antibiotic-loaded cement were first introduced by Klemm for the treatment of osteomyelitis [11]. It can deliver a high concentration of drug locally even in an avascular area and provide superior mechanical support [12], which has been widely applied to control bone infections in open fractures [13], osteomyelitis [14], and prosthetic joint infections [15]. Compared with reports on wounds in other parts of the body, there are relatively few published reports on the use of the ALBC for DSWI. VSD is an efficient drainage system and its efficiency embodies its comprehensive drainage and thorough drainage under high vacuum. It promptly and thoroughly leads seepage, pus and necrotic tissues from the drainage area out of the body to cause “zero accumulation” in the drainage area [16]. But for patients undergoing bypass surgery, the negative pressure of VSD may damage the bypass vessels. Now, VSD combined with flap metastasis has become an effective treatment for DSWI. Pitfalls of the muscle flap technique are mostly hematoma, arm strength loss, chest wall instability, infection, and pulmonary function impairment [17]. Aiming at the infection and sternal instability problems of DSWI, we apply the ALBC to our sternal reconstruction system. However, it is not clear whether the ALBC should be removed. For this patient, he had no complication. Some authors raised concerns regarding the release of cytotoxic monomers from cement including local inhibition of bone perfusion and remodeling, as well as an increased production of tumor necrosis factor possibly leading to a systemically increased bone resorption [18]. There is some unknown long-term result in the sternum that fixed with bone cement. Therefore, we decided to remove the bone cement after a period of time.

The main experience of our technique is as follows. (1) Extensive debridement followed by dead space management and adequate antibiotics administration is crucial for this patient. (2) ALBC can be adjusted to fill the wound cavity according to the size of the wound defect, leaving no dead space. After bone cement hardened, it fixed the thorax and eliminated the residual cavity. It could also be removed without difficulty if needed. (3) The antibiotics that can be used in bone cement preparation are various, in accordance with the particular sensitivity sought, providing superior mechanical support and high levels of local antibiotics. (4) VSD device is advantageous by providing continuous negative pressure to force drainage, also for eliminating residual cavity, and enhancing adhesion of subcutaneous tissue. VSD was not changed during the whole treatment, and it was removed after intermittent use for 1 week in Stage I. (5) No residual suture technique was used to fix the muscle flap, which reduced the occurrence of foreign-body infections.

## Conclusion

Although this strategy should be applied to a larger number of patients, the findings described here indicate that ALBC combined with VSD is a possible treatment option for DSWI after cardiovascular surgery, especially for those patients who are not suitable for muscle flap transfer. Future prospective, randomized controlled trials will provide a more rigorous assessment of its efficacy.

## Data Availability

As this paper is a case report, all data generated or analysed are included in this article.

## Abbreviations

LIMA: Left internal mammary artery
LADA: Left anterior descending artery
ALBC: Antibiotic-loaded bone cement
VSD: Vacuum sealing drainage
CT: Computed tomography
